# Arrhythmias May Hide a Genetic Cardiomyopathy in Left Ventricular Hypertrabeculation in Children: A Single-Center Experience

**DOI:** 10.3390/children11101233

**Published:** 2024-10-13

**Authors:** Irma Battipaglia, Nicoletta Cantarutti, Marianna Cicenia, Rachele Adorisio, Virginia Battista, Anwar Baban, Massimo Stefano Silvetti, Fabrizio Drago

**Affiliations:** 1 Pediatric Cardiology and Cardiac Arrhythmias Complex Unit, Bambino Gesù Children’s Hospital IRCCS, 00050 Rome, Italy; nicoletta.cantarutti@opbg.net (N.C.); marianna.cicenia@opbg.net (M.C.); virginia.battista@opbg.net (V.B.); mstefano.silvetti@opbg.net (M.S.S.); fabrizio.drago@opbg.net (F.D.); 2 Heart Failure, Transplant and Mechanical Cardiocirculatory Support Unit, Bambino Gesù Children’s Hospital IRCCS, 00165 Rome, Italy; rachele.adorisio@opbg.net; 3 Medical Genetic, Bambino Gesù Children’s Hospital IRCCS, 00146 Rome, Italy; anwar.baban@opbg.net

**Keywords:** left ventricular hypertrabeculation, cardiomyopathy, children, arrhythmias, sudden cardiac death

## Abstract

Background. Left ventricular hypertrabeculation (LVHT) is a myocardial disorder with different clinical manifestations, from total absence of symptoms to heart failure, arrhythmias, sudden cardiac death (SCD), and thromboembolic events. It is challenging to distinguish between the benign and pathological forms of LVHT. The aim of this study was to describe the arrhythmic manifestations of LVHT in a large group of pediatric patients and to correlate them with genetic results or other clinical markers. Methods. We retrospectively enrolled 140 pediatric patients with diagnosis of LVHT followed at our Institution from 2013 to 2023. Data regarding family history, instrumental exams, cardiac magnetic resonance, genetic testing and outcomes were collected. Most of them had isolated LVHT (80.7%); in other patients, mixed phenotypes (hypertrophic or dilated cardiomyopathy or congenital heart disease) were present. Results. Arrhythmias were found in 33 children (23.6%): 13 (9.3%) supraventricular tachyarrhythmias; 14 (10%) ventricular arrhythmias (five frequent PVCs (premature ventricular contractions), eight patients with ventricular tachycardia (VT), one ventricular fibrillation (VF)); two (1.4%) sinus node disfunctions; two (1.4%) complete atrio-ventricular blocks (AVB), three (2.1%) paroxysmal complete AVB, one (0.7%) severe I degree AVB. Three patients received an ICD (implantable cardioverter defibrillator). Comparison between LVHT patients with (33 pts) and without (107 pts) arrhythmias as regards genetic testing showed a statistical significance for the presence of class 4 or 5 genetic variants and arrhythmic manifestation (*p* = 0.037). Conclusions. In our pediatric cohort with LVHT, good outcomes were observed, but arrhythmias were not so rare (23.6%); no SCD occurred.

## 1. Introduction

Left ventricular hypertrabeculation is a heterogeneous myocardial disorder characterized by abnormal trabeculations in the left ventricular lumen and deep intertrabecular recesses [1]. According to the recent guidelines of the European Society of Cardiology (ESC) on the management of cardiomyopathies [2], LVHT cannot be considered a cardiomyopathy in the general sense, but rather a phenotypic trait that can occur either in isolation or in association with other conditions, such as left ventricular hypertrophy, dilation, systolic dysfunction or developmental abnormalities.

In recent decades, the incidence of LVHT has considerably increased due to higher sensitivity of advanced imaging techniques, both echocardiography and cardiac magnetic resonance (CMR).

However, epidemiological data may vary because of the absence of universally accepted gold standard criteria for the diagnosis of this condition [3,4,5]. Furthermore, LVHT can be a genetic disorder, with familiar inheritance or de novo mutation [6,7,8,9], or an acquired disorder such as a reversible condition in cardiovascular overload conditions (vigorous sport, pregnancy, etc.) [10,11].

In a recent meta-analysis on adult patients, the pooled prevalence estimates for LVHT were higher among cohorts diagnosed with CMR imaging (14.79%) compared with echocardiograms (1.28%). Moreover, in athletic cohorts, there was a higher pooled prevalence using both echocardiogram (3.16%) and CMR imaging (27.29%) definitions [12].

Clinical manifestations in LVHT vary greatly: from no symptoms to severe heart failure, arrhythmias, sudden cardiac death (SCD) or thromboembolic events. Children with non-isolated LVHT (mixed phenotype with hypertrophic cardiomyopathy (HCM), dilated cardiomyopathy (DCM) or restrictive cardiomyopathy (RCM)) mostly present with the symptoms that are predominant in the associated cardiomyopathy [13,14,15], and prognosis strongly depends on the prevalent phenotype.

Recently, several reviews regarding LVHT in children have shown that heart failure and arrhythmias are predictive of negative outcomes, particularly in the first year of life [16,17].

The aim of this study was to describe the arrhythmic manifestations of LVHT in a single-center pediatric population at a mid-term follow-up and to correlate them with genetic results or other clinical markers.

## 2. Materials and Methods

This is a single-center and retrospective study including all pediatric patients with LVHT followed-up at the Cardiomyopathy Clinic of Bambino Gesù Children’s Hospital IRCCS from January 2013 to June 2023.

The study protocol conforms to the ethical guidelines of the 1975 Declaration of Helsinki. Considering the retrospective nature of the analysis, our study did not require the approval of the local ethics committee according to current legislation, but a notification was sent.

Inclusion criteria were age ≤ 18 years and imaging diagnosis of LVHT: according to Jenni’s criteria, the existence of a bilayered myocardium and a noncompacted to compacted ratio > 2:1 in end-systole when using echocardiography; according to Peterson’s criteria, in CMR imaging a noncompacted to compacted ratio > 2.3 in end diastole using long-axis view.

Exclusion criteria were heart failure with LV ejection fraction < 40% at diagnosis or during follow-up and other extra-cardiac comorbidities or familiar problems which could make it difficult to follow up on the patient.

Data were collected at in-hospital follow-up evaluations and during remote-monitoring analysis.

All data were registered and retained in the hospital’s computing archives.

At first evaluation, all patients underwent collection of familiar and personal anamnesis, physical examination, 12-lead standard ECG, transthoracic color-Doppler echocardiography, and 24 h ECG Holter monitoring. CMR imaging and exercise stress tests were performed in patients ≥ 8 years old, when cooperative; blood sample was obtained for general hematological and chemical analyses and for DNA extraction. Genetic testing was performed at the Genetic Laboratories of the Bambino Gesù Children’s Hospital, IRCCS. DNA was extracted from peripheral blood with Qiagen columns (QIAamp DNA Minikit; Qiagen, Hilden, Germany) according to the manufacturer’s instructions. Next-generation sequencing (NGS) analysis was performed on genomic DNA using the Twist Custom Panels (Clinical Exome Twist Bioscience) according to the manufacture’s protocol on an Illumina NexSeq550 or NovaSeq6000 platform (Illumina, San Diego, CA, USA). Gene variants associated with cardiomyopathies and channelopathies gene were analyzed.

### 2.1. Follow-Up

Patients were followed-up on every 3, 6 or 12 months. A physical examination, ECG, echocardiography and 24 h ECG Holter were performed in all patients. Blood tests were performed if necessary, and an exercise stress test was performed in cooperative patients.

For patients with a loop recorder, a pacemaker or an ICD, remote monitoring was activated, and they were all evaluated at least twice a year.

### 2.2. Statistical Analysis

Patients with and without arrhythmic manifestations were compared according to genetic testing results, the presence or absence of associated CHD and a family history of LVHT or another cardiomyopathy. Categorical variables were compared using a chi-square test or Fisher’s exact test, as appropriate. A *p* value < 0.05 was considered statistically significant. All analyses were performed with StataSE 12.0 (StataCorp, College Station, TX, USA).

## 3. Results

### 3.1. Study Population

From January 2013 to June 2023, 140 pediatric patients were enrolled in this study (70 males, mean age 13.4 ± 5.9 years). In 46 (32.8%) patients, a family history of cardiomyopathy was found with possible different phenotypes even in first-degree relatives (HCM, DCM, ACM (arrhythmogenic cardiomyopathy) or isolated LVHT). Seventy-one patients (51%) were enrolled after a screening visit, 52 patients (37%) referred symptoms (angor, dyspnea, fatigue) or they had other cardiac or non-cardiac malformations, 17 children (12%) underwent echocardiography and subsequent diagnosis of LVHT for documentation of arrhythmias.

An isolated LVHT was diagnosed in 113 patients (80.7%), a mixed phenotype of HCM/LVHT in 4 (2.8%), DCM/LVHT with EF ≥ 40% in 10 (7.1%), an associated CHD in 13 (9.3%): 3 ASD (atrial septal defect), 6 VSD (ventricular septal defect), 1 aortic coarctation, 1 patent ductus arteriosus, 1 bicuspid aortic valve, and 1 Ebstein anomaly) (See Table 1).

### 3.2. Arrhythmias and Follow-Up

During a mean follow-up of 51 ± 19 months, arrhythmias were recorded in 33 (23.6%) children: supraventricular tachyarrhythmias in 13 (9.3%); ventricular arrhythmias in 14 (10%), sinus node dysfunction in 2 (1.4%), III degree atrio-ventricular block (AVB) in 2 (1.4%), paroxysmal III degree AVB in 3 (2.1%), and I degree AVB in 1 (0.7%).

CMR was performed in 25 (75.7%) patients with arrhythmias and, among them, one patient (4%) with frequent PVCs showed LGE (a blended inferior-junctional area of LGE).

CMR was performed in 50 (46.7%) patients with no arrhythmic events, and LGE was found in only 1 (2%) case (small area in the akinetic septal-basal-posterior wall).

No patients died during follow-up.

### 3.3. Tachyarrhythmias

Thirteen (9.3%) patients had supraventricular tachyarrhythmias:

- 4 atrioventricular reentry tachycardia;
- 1 atypical atrioventricular nodal reentry tachycardia;
- 1 atrial fibrillation;
- 3 ectopic atrial tachycardia (one was a multifocal form);
- 2 very frequent premature atrial contractions;
- 2 asymptomatic ventricular pre-excitations which were revealed to be low-risk accessory pathways by a transesophageal electrophysiology study (TE-EPS);

Fourteen (10%) patients had ventricular arrhythmias:

- 5 frequent PVCs (usually asymptomatic, monomorphic, with high arrhythmic burden at ECG Holter monitoring and variable response to exercise stress test);
- 8 sustained or non-sustained VT;
- 1 VF.

Left ventricle EF was >48% in all patients with ventricular arrhythmias at the moment of first arrhythmia recorded.

**Figure 1:**
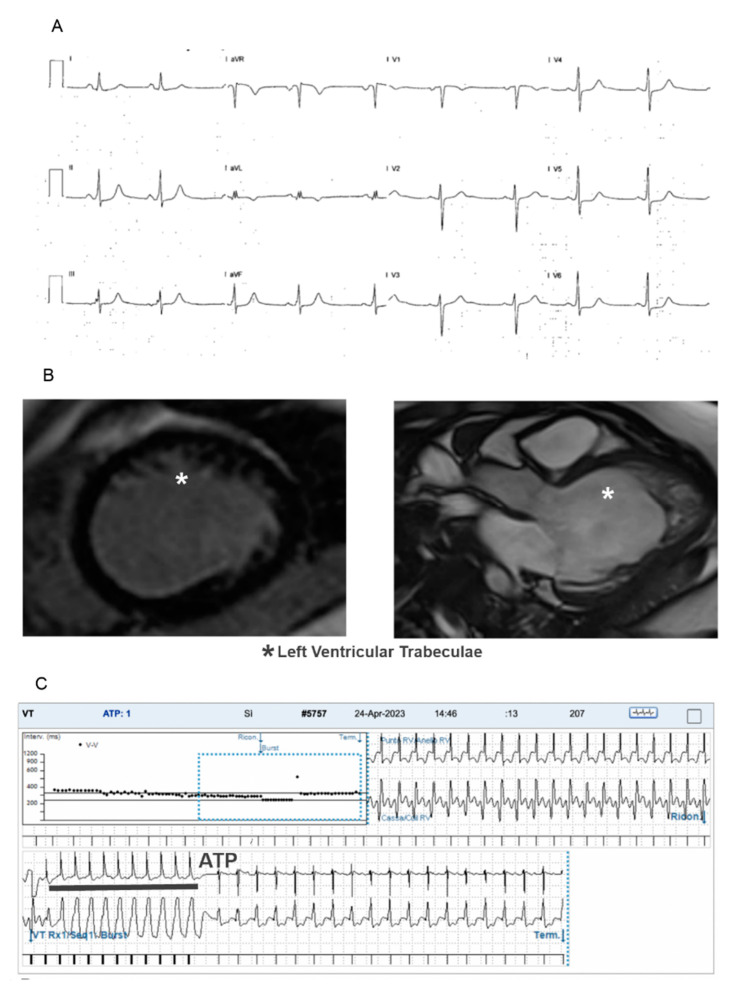
Exam results in a LVHT child with arrhythmic manifestation (familiar *PRDM16: c.370delA* genetic variant, class 4): (**A**) 12 lead standard ECG showing sinus rhythm and nonspecific ventricular repolarization abnormalities, (**B**) CMR images showing left ventricular trabeculae without LGE and (**C**) Carelink^®^ transmission showing anti-tachycardia pacing (ATP) for a ventricular tachycardia.

In three patients, an ICD was implanted. A secondary prevention implant was performed in the patient with LVHT + VSD who was resuscitated from VF and in a young child with a mitochondrial disease and history of cardiorespiratory arrest. An ICD was implanted in primary prevention in a patient with symptomatic sustained recurrent VTs. ICD follow-up recorded appropriate shocks on VTs and anti-tachycardia pacing (ATP) therapies for two of these patients (one patient implanted for secondary prevention and one patient implanted for primary prevention of SCD; see Figure 1). Patients with ventricular arrhythmias had isolated LVHT in most cases, DCM/LVHT in one case and an associated congenital heart defect in two cases (see Table 2).

### 3.4. Bradyarrhythmias

Two (1.4%) patients had sinus node dysfunction. One of these was lost at follow-up after receiving an indication to implant a pacemaker; the other one is completely asymptomatic with prevalent junctional rhythm.

Two (1.4%) patients had III degree AVB and were implanted with a transvenous pacemaker and an epicardial system, respectively.

Three patients (2.1%) had paroxysmal complete AVB, one patient (0.7%) had I degree AVB with very long PR interval (see Table 2).

### 3.5. Genetic Testing

Genetic testing was performed in 30 (91%) out of 33 arrhythmic patients and in 64 (60%) out of 107 non-arrhythmic patients.

Genetic testing revealed likely pathogenic (LP, class 4) or pathogenic (P, class 5) variants according to ACMG guidelines in 13 patients (43.3%) with arrhythmic events (see Table 3). In fact, genetic variants were reported in genes *NKX2.5* (three patients), *LMNA, PRDM16*, *NDUFA1*, *KCNJ8*, *TNNT2*, *TTN* (two patients), *SMARCD1*, 1p36 deletion, *CTNNA3* (see Table 2).

Comparison between patients with LVHT and arrhythmias and patients without arrhythmias who underwent genetic testing showed that a higher percentage (43% vs. 22%, *p* = 0.037) of patients with arrhythmias had class 4 or 5 genetic variants (likely pathogenetic or pathogenetic variants, according to ACMG guidelines).

There was a trend towards statistical significance for familiar history of cardiomyopathy and the presence of arrhythmias (*p* = 0.08). We did not find a statistically significant association between CHD/LVHT and arrhythmias (*p* = 0.49). (See Table 3).

The patients (three children) who underwent ICD implantation had positive genetic testing results (*NDUFA1*, *LMNA*, and *PRDM16*) and two of them also a positive familiar history for cardiomyopathy. One of them had a mitochondrial disease, one a mixed phenotype (DCM/LVHT) and the other had an associated VSD.

## 4. Discussion

As far as we know, this is the first pediatric study regarding LVHT concentrating on arrhythmic manifestations at mid-term follow-up, in patients with normal or only mild left ventricular dysfunction and isolated LVHT or mixed phenotypes.

Despite many studies published in recent decades, controversy remains if the finding of trabeculations in the left ventricle fulfilling criteria for LVHT is a real disease or a morphological trait [2,18,19].

Large-scale studies using echocardiography in both children and adults have estimated the prevalence of excessive left ventricular trabeculation to be between 0.02% and 0.14% [20,21] and, among children, LVHT would be the third most prevalent “cardiomyopathy” after DCM and HCM [22]. Furthermore, the incidence of LVHT has been estimated at 0.12 per 100,000 in children up to 10 years of age and 0.81 per 100,000 in children up to one year of age [23].

In a large registry of pediatric cardiomyopathies, including 98 centers in the USA and Canada over an 18-year span, 4.8% of cardiomyopathies corresponded to LVHT [24].

It is well known that LVHT can manifest as a sporadic or a familiar disease.

Genetic testing, especially in the pediatric population, is becoming more and more important to establish the possible pathogenicity of the LVHT phenotype and to predict outcomes [25,26]. With next-generation sequencing (NGS), a genetic cause is identified in up to 30% of adult cases, but in up to 44% in children [27].

In our study, genetic testing was positive for class 4 or class 5 variants according to ACMG guidelines and significantly associated with the presence of arrhythmias. This result may prove that arrhythmias may be considered a “red flag” for a genetically determined cardiomyopathy in patients with hypertrabeculation.

In most cases, LVHT is inherited in an autosomal dominant mode. Compared with adults, children more frequently have X-linked disease, a mitochondrial inherited defect or chromosomal anomalies [28]. Furthermore, genotype–phenotype correlations have been assessed. Mazzarotto et al. showed that non-truncating (transmembrane) variants in *HCN4* and structural variants in *RYR2* (genes involved in arrhythmia phenotypes) were enriched in the LVHT phenotype [29].

Clinical manifestations of LVHT can vary greatly, the most common being heart failure (46% of cases), followed by diagnosis in asymptomatic patients or as part of family screening (35% of cases) [17]. In some cases, the first clinical manifestation of LVHT can be arrhythmias that can also be fatal (SCD) and are very difficult to predict. They are not always related to left ventricular dilation or dysfunction [1], and myocardial hypertrabeculation does not explain the appearance of arrhythmias [30,31]. In fact, PVCs and VTs observed in patients with LVHT often originate from the ventricular outflow tract or from fibrosis sites far away from trabeculated areas [32]. Data from a large cohort of children, collected by Brescia et al., showed a prevalence of VTs between 0% and 38% and supraventricular tachycardia between 8% and 13%, with a particular prevalence of AVRT or EAT; SCD incidence was reported to be from 0% to 13%, with none occurring in children without previous arrhythmias or those with normal cardiac size and function [14].

Completely in line with these data, in our cohort, 10% of children had ventricular arrhythmias, 9.3% of them presented supraventricular tachyarrhythmias, and 5.7% of patients showed bradyarrhythmias.

Of note, in our study, significant ventricular dysfunction was an exclusion criterion because of its known association with a worse prognosis (also arrhythmic manifestations).

A recent review about predictors of fatal arrhythmic events in adult patients with LVHT by Bazoukis et al. concluded that symptoms, ECG anomalies (such as left bundle branch block), CMR imaging documenting ventricular dysfunction, dilation or LGE, and a non-compacted/compacted ratio of hypertrabeculated myocardium can be predictive of ventricular arrhythmias. In some patients, an electrophysiology study can be recommended for arrhythmic risk stratification [33].

Few studies on risk stratification of arrhythmias in pediatric patients with LVHT have been published: LGE at CMR imaging seems to be a predictor of ventricular arrhythmias (although not statistically significant); instead, a left ventricular ejection fraction in LVHT was not correlated with development of non-sustained VT [34]. A recent study addressed the role of ion channel gene variants and correlation with arrhythmias in a large cohort of pediatric LVHT patients [35].

Differently from the others, in this study, positive genetic testing was associated with arrhythmic manifestation in LVHT children. In fact, 43% of children with arrhythmic events had a genetic test positive for variants in known genes associated with LVHT.

This result may suggest that arrhythmias are related to the underlying disease/genetic cardiomyopathy and not to the presence of extensive trabeculation per se. These data could be useful in addressing the need of a deeper risk stratification and a closer follow-up in some patients. No other clinical or instrumental markers of increased arrhythmic risk could be clearly identified.

However, even if arrhythmias were not so rare, and three patients (2.1%) had ICD implanted, our data, resulting from an accurate follow-up protocol, showed an encouraging outcome, because no SCD occurred in our study population.

## 5. Limitations

This is a single-center retrospective study including all pediatric patients with LVHT diagnosis and preserved ejection fraction or mild ventricular dysfunction. Some patients were lost at follow-up or were sent to hospitals for adult patients after the age of 18. Our mid-term follow-up might miss the occurrence of arrhythmic events or fatal outcomes later in life. Genetic results were available in 94 out of 140 patients (genetic analysis was ongoing or not performed in 46 cases). In particular, genetic analyses were performed in 30 (91%) out of 33 arrhythmic patients and in 64 (60%) out of 107 non-arrhythmic patients. Finally, LVHT associated with a CHD was only present in 4 out of 107 non-arrhythmic patients, and this very small number (3.7%) may have determined a non-significant difference between the two groups in the incidence of a mixed phenotype (LVHT/CHD).

## 6. Conclusions

LVHT is a myocardial disorder characterized by a high variability in clinical manifestations and in arrhythmic events as well. So far, there are no clear markers to stratify risk of arrhythmias in pediatric patients with LVHT. In our study, arrhythmias were present in 33 children with LVHT (23.6%). Three of them received an ICD, and in two cases a pacemaker was necessary. No SCD occurred. The existence of a genetic variant, likely pathogenetic or pathogenetic (class 4 or 5 of ACMG guidelines), was significantly associated with arrhythmic events (*p* = 0.037), suggesting a correlation between arrhythmias and an underlying genetic cardiomyopathy in pediatric patients with excessive ventricular trabeculation. Future multicenter prospective studies finalized to identify risk factors associated with arrhythmias are desirable for the appropriate management of asymptomatic LVHT pediatric patients.

## Figures and Tables

**Table 1:** Pediatric patients with LVHT diagnosis.

Total Patients 140	Number of Patients	Other
Males/Females	70/70	Mean age 13.4 ± 5.9 yrs
***Reason for diagnosis***		
screening	71 (51%)	
symptoms	52 (37%)	
arrhythmias	17 (12%)	
Mean LV EF (%)	59.5%	
Family history of LVHT/cardiomyopathy	50 (29.4%)	
***Phenotypes***		
Isolated LVHT	113 (80.7%)	
HCM/LVHT	4 (2.8%)	
DCM/LVHT	10 (7.1%)	
LVHT + congenital heart disease	13 (9.3%)	ASD, VSD, aortic coarctation, patent ductus arteriosus, coronary sinus diverticulum, bicuspid aortic valve, Ebstein anomaly, right coronary anomaly

**Legend**: ASD: atrial septal defect; DCM: dilated cardiomyopathy; HCM: hypertrophic cardiomyopathy; LVHT: left ventricular hypertrabeculation; EF: ejection fraction; VSD: ventricular septal defect.

**Table 2:** LVHT children with arrhythmic manifestations.

*Arrhythmia*	*Pts*	*Sex*	*Phenotype*	*Family History of LVHT/CMP*	*Genetics* *(Involved Genes)*	*EF% (Median (IQR))*	*LGE at CMR*
**AVRT**	4	2 M	3 LVHT, 1 LVHT + CHD	0	1 TNNT2: c.460-1G>C (class 4) 1 TTN: c.53978_53979insA (class 4)	58% (7.3)	0
**EAT**	3	3 M	1 LVHT, 1 DCM/LVHT, 1 LVHT + CHD	2	1 NKX2.5: c.554G>T (class 4)	54% (2.6)	0
**AVNRT**	1	F	LVHT + CHD	0	0	66%	0
**PACs**	2	2 M	3 LVHT	2	1 CTNNA3: c.2638dupA (class 4)	56.3% (5)	0
**AF**	1	M	LVHT + CHD	1	LMNA: c.1201C>T (class 5)	48%	0
**VP**	2	1 M	LVHT	1	0	63.5% (8.5)	0
**PVCs**	5	3 M	5 LVHT	3	1 TTN: c.76114C>T (class 4)	63.2% (5)	1
**VTs/VF**	9	3 M	6 LVHT, 1 DCM/LVHT, 2 LVHT + CHD	4	1 LMNA: c.1201C>T (class 5), 1 NDUFA1: c.152_155delATAG (class 4), 1 PRDM16: c.370delA (class 4), 1 SMARCD1: c.592G>A (class 4), 1 Microdel. 1p36.33p36.32+ Microdupl. Xp22.33p22.32 (class 5)	59.3% (7)	0
**SND**	2	2 F	2 LVHT	1	1 KCNJ8: c.2dupT (class 5)	66.5% (10)	0
**III degree AVB**	2	2 M	2 LVHT + CHD	1	0	54% (2.5)	NP
**Paroxysmal III degree AVB**	3	3 F	3 LVHT + CHD	2	2 NKX2.5: c.554G>T (class 4)	60% (2.6)	0
**I degree AVB**	1	M	DCM/LVHT	0	0	55% (6)	NP

**Legend:** AF: atrial fibrillation; AVB: atrio-ventricular block; AVRT: atrio-ventricular reentry tachycardia; AVNRT: atrio-ventricular nodal reentry tachycardia; CHD: congenital heart disease; CMP: cardiomyopathy; EAT: ectopic atrial tachycardia; LVHT: left ventricular hypertrabeculation; NP: not performed; PAC: premature atrial contraction; PVC: premature ventricular contraction; SND: sinus node dysfunction; VF: ventricular fibrillation; VP: ventricular pre-excitation; VT: ventricular tachycardia.

**Table 3:** Comparison between arrhythmic and non-arrhythmic LVHT as regards genetic testing results (positive or negative for class 4 and 5 variants), congenital heart disease associated with LVHT and family history of cardiomyopathy.

No. Patients	Arrhythmic LVHT	Non-Arrhythmic LVHT	*p* Value
**Genetic test result positive**	13 (43% of tested patients)	14 (22% of tested patients)	**0.037**
**CHD associated with LVHT**	9 (27%)	4 (3.7%)	0.91
**Familiar history positive**	15 (45%)	31 (29%)	0.08

**Legend:** CHD: congenital heart disease; LVHT: left ventricular hypertraculation; statistical significance for *p* value < 0.05.

## Data Availability

The original contributions presented in the study are included in the article, further inquiries can be directed to the corresponding author.

## Abbreviations

AVB	Atrio-ventricular block
CHD	Congenital heart disease
CMR	Cardiac magnetic resonance
DCM	Dilated cardiomyopathy
HCM	Hypertrophic cardiomyopathy
LVHT	Left ventricular hypertrabeculation
PVC	Premature ventricular contraction
SCD	Sudden cardiac death
